# Evaluation of the European Health Information Training Programme (EHITP): results from InfAct Joint Action

**DOI:** 10.1186/s13690-022-00895-2

**Published:** 2022-05-07

**Authors:** Verónica Gómez, Mafalda Sousa-Uva, Rita Roquette, Ana Cristina Garcia, Carlos Matias Dias

**Affiliations:** 1 Epidemiology Department – National Institute of Health Doctor Ricardo Jorge, Lisbon, Portugal; 2 CHRC – Comprehensive Health Research Center, National School of Public Health, NOVA University of Lisbon, Lisbon, Portugal

**Keywords:** Health information, Health data, Evaluability, Evaluation, Information training programme

## Abstract

**Background:**

The InfAct (Information for Action) is a Joint Action of the European Commission’s 3rd Health Programme with the main goal to build an infrastructure of a health information system for a stronger European Union and to strengthen its core elements.

The InfAct Joint Action was developed along 36 months and structured in 10 work packages.

Portugal co-led the Work Package 6 (WP6) of this project, which included the development of the proposal of a flagship capacity building programme - the *European Health Information Training Programme* - and its evaluation. The evaluation objectives included: to evaluate the adequacy of the training programme to the health information needs in the European Member States; to identify possible changes regarding the participants selection process, the training activities and the pedagogical project; and to contribute to the understanding of the potential of the programme to add to available offers in learning on the topics of Public Health information, on the capacity building and behavioural changes in Public Health activities which can be attributed to the course, and of the potential of the programme to contribute to the alignment of health information criteria and procedures between the European Member States.

**Methods:**

The evaluation process was developed using an observational descriptive study design using a mixed methodological approach with both document analysis and primary data collected by questionnaires and interviews analysis. Mixed quantitative and qualitative data collection methods and analysis were used.

**Results:**

The proposal of the *European Health Information Training Programme* seemed adequate to the formative needs and capacities in line with the work performed by the InfAct project. In what concerns about its main thematic areas, it was also aligned with the areas identified in the previous formative needs and capacities mapping. The participants selection process proposed seemed, in general, adequate. The potential of the *European Health Information Training Programme* proposal to learning, capacity building and behavioral changes at work attributable to the course was considered positive, as well as the potential to the alignment of health information criteria and procedures between European Union Member States.

**Discussion:**

In general, we found high consistency between the results obtained from data collected by the techniques used. However, different suggestions for improvement were outlined by the evaluation study population.

**Conclusions:**

The proposed *European Health Information Training Programme* was a dynamic, flexible, sustainable formative programme in health information and focused on reducing inequalities.

**Supplementary Information:**

The online version contains supplementary material available at 10.1186/s13690-022-00895-2.

## Background

### Health Information training needs

Health Information is known as a comprehensive area including health data collection and analysis, definition of health indicators, health information management and translational research to promote the development of evidence-based health policies and evaluations [1, 2].

All actors involved in the field of health and public health in a society have health information needs. Health consumers, public health professionals, including medical doctors, nurses and other health care professionals, statisticians, health researchers, health managers, decision-makers, and health organizations, all need health information and this makes health information an area of universal interest [3].

According to the World Health Organization (WHO), strong country health information systems are required to provide good-quality data to support activities towards universal health coverage [4]. Thus, it is important that countries have the capacity to collect, analyse, interpret and use health data, namely, concerning diseases morbidity (prevalence and incidence) and mortality, frequency of its major risk factors, health service use and coverage and health systems performance and impact [4].

The increasing amount, diversity and availability of health information in a multidisciplinary area such as public health, makes efficient and effective use of health data and health information difficult and highlights the need for public health information management.

At European level, there have been discussions on the need for an improved European Union (EU) health information system. Different health information programmes are organised in a vertical way resulting in a fragmented and project-based EU health information system. The EU health information system lacks sustainability, coherence and comprehensiveness, which leaves us without an EU-wide public health monitoring or health system performance analysis that presents policy-oriented evidence and advices [5, 6].

Two of the main issues regarding health information in the EU are its comparability among countries, and the available training for health professionals and other stakeholders. Large differences may be found in terms of quality and, therefore, comparability of health information between and within EU Member States (MS) becomes difficult. One specific need has to do with training in health information. Health information is often trained in different courses or as modules of information systems or as part of epidemiology courses, but most of the courses are vertical with focus on one or only few topics [7].

The availability of updated and good quality health information and its use for evidence-informed policy making also varies between MS. This makes difficult to learn from each other and challenges the equity in health as poor health information and poor health tend to coincide. Thus, if a country or a group of countries have developed a good practice, mechanisms are lacking to disseminate it into EU-wide actions [5].

Therefore, the diversity of available health information, as well as knowledge and capacities to produce and use health information, contrasts between EU member states. For instance, only half of EU countries have conducted a national health examination survey so far [8].

The InfAct (Information for Action) project was a Joint Action on Health Information of the European Commission’s 3rd Health Programme, which aimed to improve the use of health data and information for a healthier Europe. Its main goal was to build an infrastructure of a health information system for a stronger European Union and to strengthen its core elements [6]. The project was launched in March 2018 and it embraces 40 partners in 28 European Union and associated countries.

The InfAct project Work Package 6 (WP6) included, among other objectives, mapping the needs, capacities and training programmes in Health Information in European Union Member States [7] and the development of a *European Health Information Training Programme* [9] based on the gaps of knowledge identified as well as its formal evaluation. Finally, WP6 included the development of a roadmap for a capacity building baseline training on health information. Such roadmap is expected to support critical areas of health information use and management in order to reduce health information inequities in EU MS and through Europe.

This paper reports the work developed to build and implement the evaluation of the proposal of the *European Health Information Training Programme* developed during the InfAct project and aims to produce recommendations for improvement that may be useful to build a roadmap for a capacity building baseline training on health information in Europe.

### Evaluation focus and evaluation objectives

The evaluation object was the proposal of the *European Health Information Training Programme* (EHITP) [9], including its pilot test, which consisted of a 35 hours teaching course, named “*1*^*st*^ *European School on Health Information*” [10].

Both EHITP and its pilot test were tasks performed in the WP6 of the InfAct Project, co-led by Portugal and Finland and were the main outcomes of this WP [6].

The evaluation process was also developed and conducted as part of WP6. The protocol for the evaluation process was based on the integration of the evaluation framework of the World Health Organization [11] and the Centers for Disease Control and Prevention Framework for Programmes Evaluation in Public Health [12].

The evaluation of the pilot *European Health Information Training Programme* comprised 4 phases: (1) engagement of stakeholders, description of the programme, and focusing the evaluation design; (2) gathering sound evidence and justify conclusions; (3) reporting of results and recommendations; and (4) incorporation of evaluation recommendations into a new version of the *European Health Information Training Programme*.

In phase 1, an evaluability assessment (pre-evaluation) was conducted based on the principles and methods of the theory of change [13]. The aims of the evaluability assessment were to describe the target of the evaluation through a logical model built with the participation of key stakeholders, and to define the focus of the evaluation. The logical model was built based on the results of a literature review and with the contributions of a workshop meeting with the main stakeholders (Supplementary Fig. 1).

The second phase of the evaluation theoretical model (gathering of credible evidence and justify conclusions), was achieved by integrating the components of the logical model of the EHITP and the adjustments needed to answer the evaluation questions. In this integration process, the Kirkpatrick’s Four-Level Training Evaluation Model (reaction, learning, behaviour and results) was also considered, given the formative nature of the evaluation object [14]. Thus, the evaluation framework of the EHITP proposal integrated the following components: formative needs and capacities; participant selection process; pedagogical project; formation, following the first three levels of the Kirkpatrick’s Four-Level Training Evaluation Model; and alignment between EU Member States.

The seven evaluation objectives of the proposal of the *European Health Information Training Programme* (EHITP) were thus defined as: (1) to evaluate the adequacy of the EHITP to the health information needs in the European Member States; (2) to identify possible changes to the EHITP, regarding to the selection process of the trainees and the training activities and the pedagogical project; (3) to contribute to the identification of potential main EHITP outputs through the analysis of the trainees’ attendance during the *1st European School on Health Information*; (4) to contribute to the understanding of the potential satisfaction of the EHITP participants through the satisfaction analysis expressed by the trainees and the lecturers at the *1st European School on Health Information*; (5) to contribute to the understanding of the potential of the EHITP to learning, capacity building, and behavioural changes at work, through the perceptions of the participants in the *1st European School on Health Information*; (6) to contribute to the understanding of the potential of the EHITP to the alignment of Health Information criteria and procedures between EU Member States through the perceptions of the EHITP authors and of the participants in the *1st European School on Health Information*; (7) to identify successful and unsuccessful areas or issues in the EHITP proposal and in the *1st European School on Health Information* that can help EHITP future improvement or adequacy.

## Methods

### Evaluation study design

The evaluation was performed through an observational descriptive study using a mixed methodological approach with both document analysis and primary data collected by questionnaires and analysis of semi-structured interviews. Mixed quantitative and qualitative data collection methods and analysis were used.

**Fig. 1:**
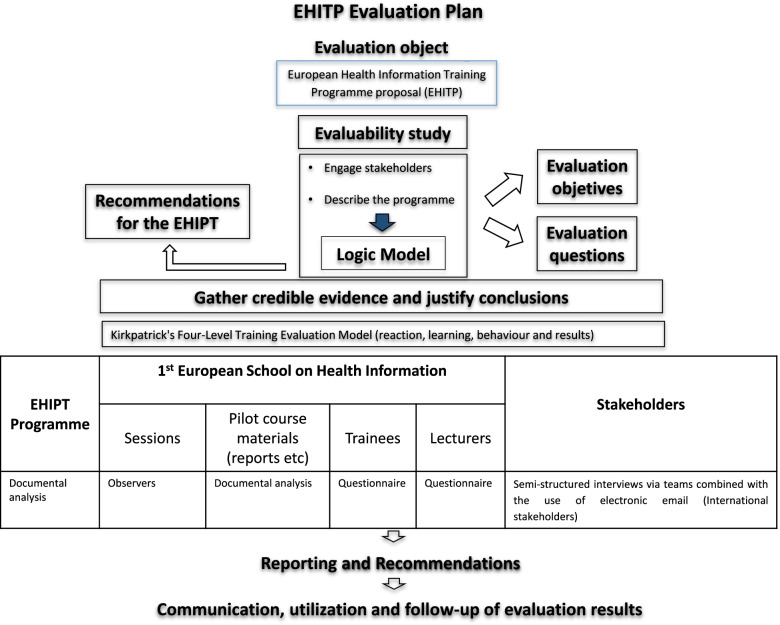
Diagram of the *European Health Information Training Programme* evaluation plan

The design of the evaluation of the EHITP proposal was defined according to the evaluation questions, the evaluation objectives and the results of the evaluability assessment, and to the evaluation model, evaluation framework and evaluation object. The EHIPT evaluation plan is summarized in Fig. 1.

### Study population

The study population of the first European School on Health Information included the trainees (*n* = 23) and the lecturers of the pilot course (*n* = 16), InfAct project coordinators (*n* = 2), coordinators and members of the InfAct WP6 (*n* = 6) and other co-authors of the EHITP (*n* = 4).

### Material, sources and data collection techniques

Data were collected using three techniques: document analysis (secondary data) based on the material made available by the coordinators of the EHITP; two questionnaires specifically built for the evaluation (one for the trainees and the other one for the lecturers of the pilot course); and semi-structured interviews with the coordinators and authors of the EHITP.

Data were collected according to the components of a measurement matrix specifically designed for the evaluative study (Table 1). For each component of the evaluation framework and corresponding evaluation questions, the measurement matrix presents a series of indicators and criteria, with the aim of converting concepts into specific and measurable sections.

**Table 1:**
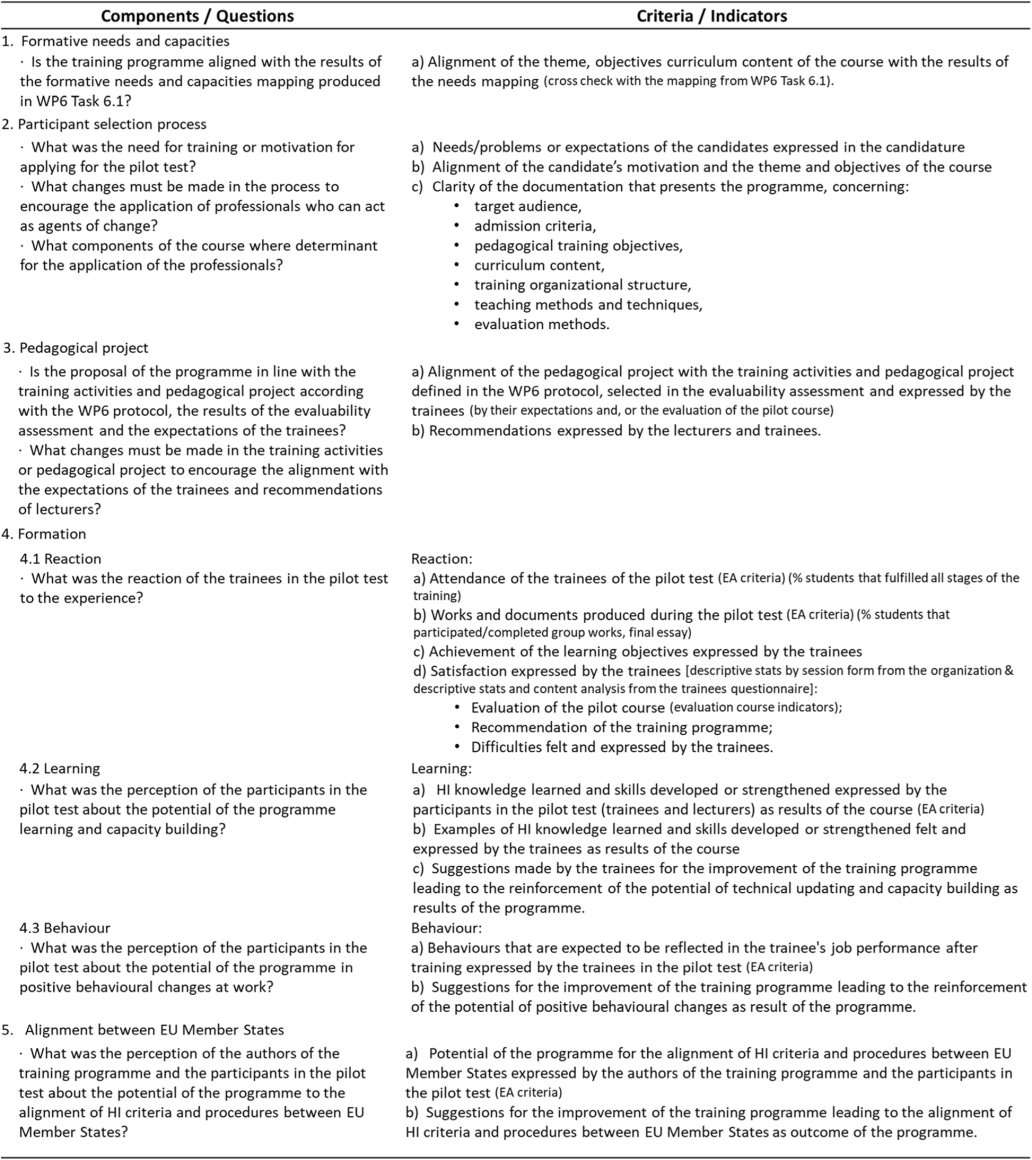
Measurement matrix

Document analysis (secondary data) was based on the material made available by the coordinators of the EHITP, which included the EHITP; documentation concerning the pilot course (participants’ application forms, the pilot course announcements, the booklet of the course, the satisfaction surveys and other course evaluation forms); and other written documents or communications between pilot course coordinators and candidates or pilot course participants.

Two questionnaires were specifically designed for the evaluation: one to be answered by the trainees and the other one to be answered by the lecturers of the pilot course. They were self-administered questionnaires, anonymized with regard to the identity of the participants. The questionnaires were distributed and answers collected by computer using REDCap [15] (Research Electronic Data Capture), preserving the anonymity of the participants’ identity. Both questionnaires included both closed and open questions, which targeted the following components of the measurement matrix: participant selection process, pedagogical project, formation and alignment between EU Member States. Variables for a brief socio-demographic characterization of the participants were also included. Both questionnaires were pre-tested by health professionals and university professors and the suggested corrections and improvements were made prior the application.

The semi-structured interviews were conducted using a script specifically designed for the purpose, based on the measurement matrix. The script aimed to define the content and to guide the interview. However, maximum flexibility was sought to identify the components that required further exploration and development. Open questions were asked to identify the perceptions of the coordinators and authors of the programme regarding the component alignment between EU Member States of the measurement matrix. A brief socio-demographic characterization of the interviewees was also included. All data were anonymized with regard to the identity of the participants. The individual interviews were carried out using Microsoft Teams (on a date agreed between the researcher and the interviewed), after the acceptance by each participant of the invitation previously sent by e-mail and having also given written consent for the use of the data in this research. Collected data were transcribed manually into text document.

Both quantitative and qualitative data analyses were performed. Data collected from the answers to closed questions of the questionnaires were analysed using frequencies distribution. The answers to open questions of the questionnaires and interviews, and data collected from document analysis were subjected to thematic analysis. A matrix of analysis categories was built from the measurement matrix to help data analysis (Supplementary Table 2).

## Results and discussion

### Participants

Of the 23 trainees invited to answer the questionnaire, 14 (60.9%) participated. Regarding the lecturers, 16 were invited to answer the questionnaire and 9 participated (56.3%). Trainees who answered the questionnaire were predominantly females (92.9%), with a mean age of 37 (minimum = 24; maximum = 74) and were predominantly medical doctors (28.6%).

Lecturers who answered the questionnaire were in equal number females and males (50% males and 50% females), with a mean age of 47 (minimum = 28; maximum = 75) and were predominantly medical doctors (28.6%).

Of the total number of stakeholders invited to the interview (*n* = 12), 11 participated.

### Formative needs and capacities

The results of the evaluation showed that the proposal of the *European Health Information Training Programme* is adequate to the formative needs and capacities of both trainees and lecturers in line with the work performed in WP6 Task 6.1 of the InfAct project [7], highlighting the importance of updating the needs assessment over time.

The main thematic areas were also aligned with the areas identified in the formative needs and capacities mapping produced in WP6 Task 6.1 of InfAct, such as: data analysis and interpretation; interoperability of data sources; transfer from data to policy; data collection, sources, metrics and indicators; and data privacy and ethical issues [7].

### Participant selection process

In general, trainees, lecturers, and stakeholders expressed a positive perception regarding the participant selection process of the *1st European School on Health Information*.

All the trainees that responded to the questionnaire (*n* = 14) considered the application criteria of the course appropriate but made some suggestions. The most expressed needs, problems, or expectations of the candidates for the *1st European School on Health Information* that motived the candidature were: acquisition or deepening of knowledge in health information, especially for reasons related to the professional activity; knowledge transfer and teaching in the scope of health information or health information systems; and health information research. Strengthening the networking was also an expectation frequently expressed. The most common themes or topics pointed by the trainees where they expressed the need to deepen knowledge were: “*health data*”; “*health information systems*”; “*health examination surveys*”; “*data collection and data sources*”; “*security* and *privacy issues*”; “*the validity of the data and the interoperability*”; “*transfer to policy making methods”.*

Lecturers considered the participant selection process of the course positive and also made some suggestions and recommendations: *“Advertising the course to national experts and students”; “More people wanted to participate than places were available ...More places should be made available.”; and “Wider and earlier publicity for the programme”.* The lecturers also recommended changes in the participant selection process: *“[it may be] more profiles of participants”; “May be several editions for: a) residents or students, b) professionals, etc”; “Probably have well fractioned audience to tackle different issues: young professionals who need mentoring vs exchange programmes for seniors”; “The courses should be adapted to a cohort of specialists; course must be conditions/circumstances dependent.”; “In new editions it would be probably important to introduce new/more criteria to the selection of participants (individual training or work areas/fields)”; “I would not strict too much the selection process”.*

In general, the main suggestions and recommendations regarding the participant’s selection process were a wider and earlier call for participation, involving eventually the social media, and the inclusion of more profiles of participants and different pedagogical projects accordingly*.*

Regarding to the alignment of the candidate’s motivation and the theme and objectives of the course, themes and topics of the *1st European School on Health Information* curricular programme were coherent with most of the expressed needs and motivations.

All the trainees (*n* = 14) that answered the questionnaire considered the materials made available in the booklet of the course sufficiently clear (educational training objectives, curriculum content, organizational structure of the training, and teaching methods and techniques).

### Pedagogical process

In general, the EHITP proposal is aligned with the WP6 InfAct protocol [5], as well as with the results of the evaluability assessment and with the expectations of the trainees of the *1st European School on Health Information*.

Alignment between the pedagogical project and the expectations of the trainees of the *1st European School on Health Information* was found. However, the participants suggested: to increase the time of the course; to timely share the materials; a better communication between all the participants and coordinators; and to deepen the vocational character of the course and specific thematic areas (General Data Protection Regulation - GPDR -, interoperability and methodological approaches based on epidemiology and public health).

The stakeholders’ interviews results about the quality of the pedagogical project were in general consistent, especially with regard to the quality and adequacy of the lecturers and sessions (interconnected and not overlapped).

### Training

The evaluation of the reaction of the trainees in the *1st European School on Health Information* to the course was generally positive.

Regarding the attendance of the course, 19 of the 23 trainees participated in all or all except one (90%) of the sessions held. From the trainees that responded to the questionnaire: all considered that in general the learning objectives were achieved; 11 out of 14 (78.6%) considered that the course contributed to learning and/or improving their technical execution skills; 11 out of 12 (91.7%) considered that the course contributed to learning and/or improvement in health information; and 11 out of 13 (84.6%) admitted advising to replicate the experience to other potential trainees. Five trainees referred having experienced difficulties during the course, especially concerning the time available to assimilate an important amount of new information or issues considered more complex or difficult.

According to the document analysis of the assessment surveys reports of the course, the trainees’ satisfaction was consistent with the results of this evaluation. Through a scale ranging from 1 (not suitable) to 5 (fundamental), from those that answered (more than 50% in all sessions) more than a half considered the sessions very suitable (value 4) or fundamental (value 5).

The examples given by trainees to illustrate the learning and the improvements expressed include: having acquiring or deepening knowledge in specific topics in the health information domain (for instance, health surveys, health programmes, health information systems, data sources, data collection, comparison of different countries strategies, data linkage, GDPR and ethics understanding, interoperability, data translations, development of a public health report); and exchange of “*knowledge and skills with others*”; improvement of “*knowledge of the European data landscape and how to navigate it*”; “*better critical thinking*” or “*better work in a European team*”.

In general, the trainees acknowledged a positive evolution in their professional activity as an outcome of their participation in the course, namely, in the following areas: integration of scientific and technical knowledge (9 positive answers in 11); technical execution capabilities (7 positive answers in 10); use of technical language and terminology (8 positive answers in 11); work capacity towards greater productivity (10 positive answers in 11); professional motivation: (9 positive answers in 11); and acquisition of new professional skills during the course (6 positive answers in 10).

All lecturers who evaluated globally the course (*n* = 8) considered the initiative as positive: “*It had to be virtual and, in spite of that, the overall opinions of the participants about the contents and their learning process was satisfactory*”; “*Generically, it involved students from almost all EU countries that were interested and developed interesting work during the course sessions*”; “*On the day which I delivered a lecture and facilitated a discussion group, all the participants had a very positive attitude and engaged very well with each other and with the lecturers*”; “*The course was a success and it delivered valuable knowledge and experience to participants who were interested in health information*”; “*As a pilot course I wasn’t sure about the response from the students to the contents, but it turned out really well*”. However, lecturers gave some suggestions for an improved communication at all levels, for instance: “*Communication to lecturers … could be improved. Responding to emails would be my number one. Providing clarity times schedules a good second.*”

### Alignment between EU Member States

The perception of trainees, lecturers and interviewees on the potential of the EHITP proposal to contribute to the alignment of health information criteria and procedures between EU Member States was in general positive, being admitted the homogenization of capacity building and the alignment of criteria and procedures with the replication of the courses, and a potential positive impact on global public health development. The results of the document analysis were also consistent.

### Limitations

In this study it was not feasible to measure the impact of the EHITP, not even in an exploratory way, mainly due to the short time between the only formative experience performed up to now (the *1st European School on Health Information*, as the pilot test of the EHITP) and the evaluation data collection. Therefore, despite the fact that the evaluation design is based on a logical model, the attribution of results and impact cannot be addressed. Although the European and national contexts are integrated in the logical model of the EHITP, it was not possible to consider its effects in the discussion of the evaluation results.

Due to the pandemic, all phases of the evaluation were done remotely, which may have, to some extent, hindered part of the qualitative approach, as it was not possible to conduct the interviews in person (although it was through video calls). Another limitation of this evaluative study is related to the data collection technique applied to the trainees and lecturers - online questionnaire - often associated with a low response rate, despite reminders. This may call into question the representativeness of the study population. However, the high consistency with the results obtained from data collected by the other techniques, leads us to admit a minimal effect of that fact.

## Conclusions and recommendations

The proposed *European Health Information Training Programme* was a formative programme in health information, dynamic, flexible, sustainable, and focused on reducing inequalities, as stated in InfAct project protocol. The global evaluation was positive concerning all components of the logical model, including the documentation that presented the course; the pedagogical project; the learning, capacity building and potential to behavioural changes at work attributable to the course; and the alignment of criteria and procedures in health information between the EU MS.

The main specific recommendations of the evaluation of the EHITP proposal are aimed especially at strengthening some components of the proposal, in view of future courses/ training activities within the scope of the EHITP. These can be summarised as follows: a) a special note to the adequacy of the participant selection process regarding the time of the application period and the profile of the candidates; b) reinforcement of the importance of the regular update of the health information needs assessment and use of the results; c) sustaining the preference for courses with modular curricular programmes and a diverse curricular contents; d) insistence on an in-depth approach to curriculum content related to thematic areas considered at the time of particular relevance, such as the General Data Protection Regulation (GDPR) and ethical issues, interoperability, and methodological approaches based on epidemiology and public health; e) improvement of the communication tools between all the participants in the programme – coordinators, lecturers, and trainees; f) use of the Distributed Research Infrastructure on Population Health (DIPoH) when possible and adequate; g) development of impact evaluation studies of the EHITP.

The final recommendation is the incorporation of the specific recommendations in a new version of the *European Health Information Training Programme*, and its use in the development of the Roadmap for the Capacity Building Programme in Health Information planned for Task 6.4 of the InfAct Joint Action.

Regarding future research concerning this topic, we suggest that more in depth methods related with remote learning should be explored. The COVID-19 pandemic has revolutionized remote training and it seems to show that can be easily implemented with a few basic tools. Within the field of public health, we believe that future investigations should take into account the most up to date training theories in fully remote learning models. Given the time span that is required, future investigations should contemplate the impact of this program to measure the attribution of results and impact.

## Supplementary Information

**Additional file 1.**

## Data Availability

Data generated or analyzed during this study are included in this published article [and its supplementary information files], further information is available from the corresponding author on reasonable request.

## Abbreviations

DIPoH: Distributed Research Infrastructure on Population Health
EHITP: European Health Information Training Programme
EU: European Union
GPDR: General Data Protection Regulation
InfAct: Information for Action
MS: Member States
REDCap: Research Electronic Data Capture
WHO: World Health Organization
WP6: Work Package 6
