# Supplementary material for: Evaluation of the European Health Information Training Programme (EHITP): results from InfAct Joint Action
Source: Arch Public Health. 2022 May 7;80:133. doi: 10.1186/s13690-022-00895-2 (PMC9076499; doi:10.1186/s13690-022-00895-2)
Supplement: Supplementary file 1 — Additional file 1. [file 13690_2022_895_MOESM1_ESM.docx]

## Suplementary Figure I - Logical model of the *European Health Information Training Programme*

**Logical model of the Flagship Capacity Building Programme, according to the results of the evaluability study**

^1^ Namely within the context of health information policies in Europe and European data protection regulations

^2^ In particular, in the context of national health policies, national health information policies and national data protection policies

Suplementary Table I - Matrix of analysis categories
